# Sleep Deprivation and Advice Taking

**DOI:** 10.1038/srep24386

**Published:** 2016-04-25

**Authors:** Jan Alexander Häusser, Johannes Leder, Charlene Ketturat, Martin Dresler, Nadira Sophie Faber

**Affiliations:** 1 Department of Social Psychology, Justus-Liebig University Giessen, Germany; 2 Institute of Psychology, University of Bamberg, Germany; 3 Institute of Psychology, University of Hildesheim, Germany; 4 Max-Planck Institute for Psychiatry, Munich, Germany and Donders Institute, Radboud University Medical Centre, Nijmegen, The Netherlands; 5 Department of Experimental Psychology, University of Oxford, Oxford, United Kingdom

## Abstract

Judgements and decisions in many political, economic or medical contexts are often made while sleep deprived. Furthermore, in such contexts individuals are required to integrate information provided by – more or less qualified – advisors. We asked if sleep deprivation affects advice taking. We conducted a 2 (sleep deprivation: yes vs. no) ×2 (competency of advisor: medium vs. high) experimental study to examine the effects of sleep deprivation on advice taking in an estimation task. We compared participants with one night of total sleep deprivation to participants with a night of regular sleep. Competency of advisor was manipulated within subjects. We found that sleep deprived participants show increased advice taking. An interaction of condition and competency of advisor and further post-hoc analyses revealed that this effect was more pronounced for the medium competency advisor compared to the high competency advisor. Furthermore, sleep deprived participants benefited more from an advisor of high competency in terms of stronger improvement in judgmental accuracy than well-rested participants.

Last year’s overnight EU summits deciding on last-minute financial rescue packages for Greece give the impression that it is becoming a tradition to make important decisions under acute sleep deprivation (SD). Many political and economic decision-makers have been faced with making judgments while suffering from insufficient sleep, even before SD had reached the status of a “public epidemic^1^”. At the same time, as in the case of EU summits, these judgments are typically not made in a social vacuum: decision makers are required to integrate a wealth of information provided by advisors such as experts, lobbyists, or consultants. Recently, we have argued that SD alters judgment and decision-making in social contexts, since it is very likely to have social effects beyond its effects on individual functioning^2^. While individual functioning and SD has received considerable attention in the psychological literature^3^, the social aspect has not. One important social aspect in judgment is the role of advice, and the study reported here is the first to examine about whether SD affects advice taking.

## Sleep deprivation and judgment

It is well documented that even one night of total sleep deprivation detrimentally affects a broad range of judgments and decisions^4^. This applies particularly to decision tasks that are low in complexity and high in monotony, as well as to more complex task environments^5^,^6^. The negative effects of SD on judgment and decision-making are likely to be rooted in SD’s negative effects on their underlying cognitive functions: a meta-analysis of studies examining cognitive effects of SD found that lack of sleep had a negative impact on accuracy and speed in diverse cognitive functions, with most robust effects for attention and working memory^3^.

One interesting question arises from these findings of performance deficits due to SD: To what extent are individuals aware of those deficits? This is particularly important with respect to the question of how we can utilize advice when being sleep deprived, since advice taking could help individuals to compensate for those deficits. No study has ever examined the effects of SD on advice taking. However, there is some evidence that allows speculation about possible effects. Studies on the ability to self-monitor cognitive performance under SD found that performance declines were mirrored in confidence ratings^7^,^8^. This finding indicates that SD did not impair the metacognitive ability to accurately evaluate one’s own performance and cognitive ability. In general reduced self-confidence in turn has been found to increase advice taking^9^.

Further suggestions on why and how SD may affect advice taking come from research showing that SD increases susceptibility to the influence of others^10^. SD has been shown to reduce self-regulatory control and it has been proposed that this effect might be mediated by impaired executive functions^11^,^12^,^13^. Reduced self-regulatory control in turn increases responsiveness to social influence, such as compliance, persuasion or conformity^14^. Hence, we argue that due to lower confidence and higher susceptibility to social influence, SD increases advice taking.

## Judge Advisor Systems

Sniezek and Buckley^15^ introduced the Judge Advisor Systems (JAS) as an experimental paradigm to systematically study social influence related to consultation and advice taking. A JAS is an (often computer mediated) experimental environment in which one person – the judge – has to make a judgment after receiving advice from a (typically non-present and unknown) advisor. JAS examine the degree to which the judge integrates or discounts the advice in his own judgments. Although the main focus of judge-advisor research lies on the effects of advice taking (particularly advice under-utilization, so-called egocentric discounting) on judgment accuracy and decisional confidence^16^, a large – yet growing – body of empirical studies tested potential moderators of advice taking. For example, it has been shown that individuals are more receptive to good advice compared to bad advice^17^ and paid advice is used more than free advice, even if it is of the same quality^18^.

Recently, research also started to account for extraneous factors that could influence advice taking – that is, variables that are not an inherent element of the JAS or the decision making situation per se. For example, it has been found that experimentally induced anxiety prior to the judgmental task promotes advice taking. This effect was mediated by lowered self-esteem^9^. In a similar vein, incidental gratitude leads to more advice taking, whereas incidental anger results in decreased advice taking^19^. SD’s high prevalence in the working world, for example, in the medical context^20^,^21^, points to the possible benefits of a better understanding of how advice might counter some of SD’s negative effects.

## The present study and hypotheses

The present study aims to test whether or not SD has an effect on advice taking. We employed a laboratory-based experimental JAS using a within-subject manipulation of advisor competency. SD was manipulated between subjects, hence we compared a sleep deprived (24h) group of participants to a control group with a night of normal sleep. We predicted higher advice-taking in sleep deprived individuals compared to those with a normal night of sleep (Hypothesis 1). Furthermore, in line with earlier research^17^, we predicted that advice from a highly competent advisor is generally more relied upon than advice from a medium competent advisor (Hypothesis 2). In a more exploratory manner we will examine potential interactions of SD and advisor competency as well as effects of SD on judgment accuracy.

## Materials and Methods

### Participants and design

Ninety-six undergraduate students (32 male, 62 female, 2 n/a, mean age = 25.6 years) were randomly assigned to the experimental conditions of a 2 (sleep deprivation: 24h without sleep vs. regular sleep) ×2 (competency of advisor: medium vs. high) mixed design, with the first factor varying between participants and the second within them. Sample size was determined by a statistical power analysis using GPower 3.1.7. In our study we expected small to medium effects (f = 0.2) using Cohen’s criteria^22^. With an alpha = 0.05 and power = 0.90, the projected sample size needed with this effect size is approximately N = 68 for the simplest between and within group comparisons. Thus, our proposed sample size of N = 96 will be more than adequate for the main objectives of this study and should also allow for expected attrition and for additional subgroup and mediation analysis. No data-collection stopping rule was applied and no analyses were run before data collection was completed. Individuals reporting working late hours regularly, chronic sleep problems or psychiatric illness were excluded from participation. The experiment was approved by the local ethics committee of the University of Hildesheim (Germany), and the study was carried out in accordance with the approved guidelines. Informed consent was obtained from all participants.

### Procedure

Two days prior to each experimental session, participants were equipped with actigraphs (GT3X-BT Monitor^®^) and sleep diaries to check for adherence to the experimental protocol. Actigraphy is a method established in sleep medicine to monitor activity, rest and sleep cycles, using accelerometers attached to the wrist of the participants^23^. Since there is some evidence that chronotype might be related to the ability to cope with extended wakefulness^24^, making evening types less prone to negative effects of SD, we included a single-item self-report measure from the Munich Chronotype Questionnaire^25^ in the sleep diaries (seven-point Likert scale, 0–6 “I consider myself as an: extreme morning type (0) – extreme evening type (6)”). Participants in the SD condition were instructed to get up at 8 am the day before the experimental session, to stay awake until coming to the laboratory in the evening (i.e., no naps during the day), and to abstain from caffeine in any form. Participants arrived at the laboratory at 12 am and stayed awake all night. During the night, research assistants were present to ensure that nobody fell asleep. Compliance was further checked using actigraphy data. Following a standard procedure in SD research^26^, participants were allowed to spend the night at their own discretion, with watching movies, reading, or listening to music. During the night beverages and snacks were provided, but participants were not allowed to consume caffeine or any other stimulants. At 7am, participants were served breakfast (i.e., cereals, bread, fruit of their choice) and started their experimental tasks at 8am. Participants in the control condition were instructed to get up at 8am on the day of testing and to arrive at the laboratory immediately before starting their session at 10am. These participants were instructed to sleep normally during the two nights preceding the experimental session, which was controlled by actigraphy. Although the small difference in timing of our experimental sessions might add some noise, it clearly seemed the lesser of two evils to us: control condition sessions could not be scheduled earlier, to ensure that the participants’ sleep duration was not affected by the study (i.e., participants had to get up early). The SD condition sessions, on the other hand, could not be scheduled later, to make sure we induced 24 hours of SD, not more.

Eight to twelve individuals participated in each session. All participants gave written consent for participation and were fully debriefed after the experimental session. Participants were told that they could stop the experiment anytime (which nobody did). As monetary compensation, participants received €20 as a flat fee. In the SD condition, participants were paid an additional €60 for their willingness to stay awake all night.

### Judge-advisor task

Participants were seated in front of computers that were arranged in separate cubicles to seclude them from the view of others. First, participants filled in a questionnaire on mental fatigue. The scale consisted of five items (e.g., “At the moment I am reacting slower than normal”) on a seven point Likert scale (0 = not at all; 6 = absolutely) and showed very good internal consistency (α = 0.92). Afterwards, participants completed 30 trials of a judge-advisor task. Participants were told that the task was a test of their ability to correctly estimate airline distances between pairs of European cities (e.g., Paris–Amsterdam, Dublin–Helsinki)^27^. Further, they were told that they would be allowed to use advice in form of recorded estimates by other participants who had completed the task earlier (the advisors). On each trial, they should provide a first (initial) estimate, after which they would receive advice. Participants were then asked to give a second (final) estimate for the pair of capital cities in the respective trial.

Two different advisors were used to operationalize the within-subject factor of advisor competency: one advisor was of high, the other advisor of medium competency. Participants were told that 100 individuals completed all trials of the estimation task in an earlier experiment and that these individuals were ranked according to the accuracy of their estimates. A rank of 1 indicated that this person gave the most accurate estimates; a rank of 100 indicated that the individual provided the least accurate estimates in that sample. Using a bogus random generator, participants drew two individuals from this pool of potential advisors and were told the rank of these two advisors. All participants first drew “Advisor A: rank 52” (medium competent) followed by “Advisor B: rank 7” (highly competent). (Note, however, that we did not use the labels “highly competent” and “medium competent” at any time during the task, in order to avoid biasing the participants.) The estimates of Advisor A deviated from the true flight distances by 55% (range: 51–60%), whereas the estimates of Advisor B deviated by 16% (range: 10–19%). In 15 trials for each of the two advisors, participants received advice from Advisor A and Advisor B, respectively. In each trial, after their initial estimate participants were told which of the two advisors provided the advice. The advice was provided in a written form of the kind “Advisor A estimated 500 km”. Both advisors gave advice that sometimes underestimated and sometimes overestimated the true distances, with 47% being underestimations. Trials were presented in a random order.

Participants were asked to work as accurately as possible on the task. They worked in a self-paced manner, there was no external time limit for the overall task, and the 30 trials were presented in one block without any breaks. The judge-advisor task was programmed and presented using z-tree (Zurich Toolbox for Readymade Economic Experiments)^28^.

### Data preprocessing

*Advice taking*. To quantify the degree to which participants integrated the advice into their final estimates, we employed a weight of advice (WOA) measure commonly used in advice taking research^16^. The WOA indicates the degree of use of advice and is defined as the absolute difference between the final estimate and the initial estimate as proportion of the absolute difference between the advice and the initial estimate.

WOA =| (final estimate_xi_ - initial estimate_xi_) |/| (advice_x_ - initial estimate_xi_) | with *x* referring to each city pair and *i* to each individual’s estimate. The WOA measure ranges from zero (indicating complete discounting of advice) to one (indicating that the final estimate is equal to the advice). For example, when the initial estimate is 400 km (e.g. for the flight distance for Paris-Amsterdam), the advice is 500 km and the final judgment is 450 km, a 50% shift (WOA = 0.5) occurred. In other words, the judge weighted the advice to 50%.

Although WOA-values >1 are possible in theory (when the final estimate overshoots the advice), this occurs only infrequently (in about 6% of the cases reported in the literature^29^). Since there is no reason to assume that the judge should weigh the advice to more than 100% (from a conceptual perspective, it could even be argued that this is impossible), we set all WOA-values >1 to 1, as it is common practice in judge-advisor research^29^,^30^,^31^. Out of a total of 2880 (30 × 96) WOA-values, 236 (8%) were corrected accordingly. The corrected WOAs were used as indicators of advice-taking.

#### Accuracy of estimates

As an indicator of the accuracy of estimates, we calculated the mean absolute percent error (MAPE). This represents the deviation of the estimates from the true distance^27^. The MAPE-score is defined as the mean of | (estimate_xi_ - true distance_x_)/true distance_x_ | *100 with *x* referring to each city pair and *i* to each individual’s estimate. Hence, for each trial’s initial and final estimate, we calculated the absolute value of the difference between the estimate and true distance proportional to the true distance multiplied by 100. For example, if the estimate is 400 km for the flight distance for Paris-Amsterdam (true distance: 429 km) the MAPE-score is 7, indicating a 7% deviation from the true distance. Then we calculated the means of the trials (overall for all 30 trials; as well as for the 15 trials with the highly and the medium competent advisor, respectively). This was done for the initial and the final estimates.

For our study it was irrelevant whether the deviations from the true air-line distances were over- or underestimations, so as a result we used absolute values. The lower boundary of the MAPE-scores was 0, with lower scores indicating higher accuracy. There was no given upper limit for MAPE-scores. The observed range of the MAPE for initial estimates was 0–98. The observed range for the MAPE of final estimates was 0–84.

## Results

### Sleep before testing

To examine sleep duration in the control condition, we analyzed actigraphy data using a standard sleep scoring algorithm^32^ and validated it with sleep diary data. Participants in the control condition slept on average for 396 min (*SD*: 63.26; range = 282 – 545 min). For two participants in the control condition actigraphy data was missing. For those two participants we used the sleep diary data and included them in the analyses. None of the participants in the SD condition fell asleep.

### Mental fatigue

The 46 participants in the SD condition reported higher mental fatigue (*M* = 4.33, *SE* = 0.17), compared to the 50 participants in the control condition (*M* = 1.73, *SE* = 0.16), *t*(94) = 11.32, *p* < 0.001, d = 2.32, 95% CI [−3.05, −2.14].

### Advice taking

We used R^33^ and *lme4*^34^ to perform a linear mixed effects analysis of the effects of advisor competence and sleep deprivation on the advice taking (WOA scores). In the final model, we entered advisor competence and sleep deprivation as fixed effects. As random effects we assumed random intercepts for subjects and trials, but we did not assume random slopes. We assumed a random intercept model, because deviations from the grand mean (i.e., WOA under different conditions) were of interest and we had no predictors on level 1, which would suggest random slopes. P values were obtained using the *lmertest* package^35^. The intercept in the baseline model represents the grand mean. Intercepts in the subsequent models always represent the mean of the reference category, which assumes a value of 0 for all entered predictors. Here this is the control condition for the predictor condition and the medium competent advisor for the predictor advisor competence.

First, we checked the intra-class correlation (ICC) for the baseline model. This model predicted WOA intercept (the grand mean of WOA) with a random intercept for trial and subject. The ICC for the WOA for subject as a grouping factor for each participant was .14 due to random effects, suggesting a multi-level model as appropriate. The ICC is based on the variance of the random effects and the residual variance in model 0. The estimates and results for all models are depicted in Table 1.

To the baseline Model 0 we added the factor condition (control vs. SD) as fixed effect resulting in Model 1. Model 1 showed that introducing the factor condition significantly added to the prediction, *χ*^2^(1) = 9.73, *p* = 0.002.

Model 2 added the competency of the advisor as a fixed effect to model 1 and led to a decrease of variance for the random intercept of trial, since the advisor competence explained variance between trials. Model 2 yielded a significantly better fit than model 1, *χ*^2^(2) = 389.85, *p* < 0.001 and two main effects for condition and advisor competence.

**Figure 1:**
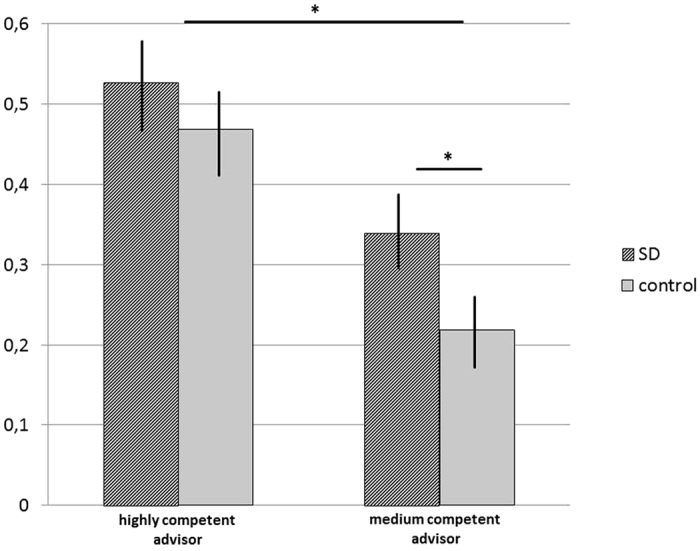
WOA-values by competency of advisor and experimental condition. Data presented as group means ± SEM. *p < 0.05.

Model 3 allowed for an interaction of condition and advisor competence and showed a significantly better fit than model 2, *χ*^2^(1) = 6.70, *p* = 0.0097. In sum, our analyses supported Hypothesis 1, by revealing that participants in the SD condition adjusted their final estimates towards the advice to a higher degree, compared to participants in the control condition (43% vs. 34%). In line with Hypothesis 2, adjustment was stronger in the highly competent advisor trails compared to the medium competent advisor trails (50% vs. 28%). To disentangle the interaction between condition and advisor competency, we calculated post-hoc simple effects. These analyses showed that the difference between SD condition and control condition was more pronounced in the medium competent advisor trials, compared to the highly competent advisor trials (medium competent advisor: *M*_*SD*_ = 0.34, *SE*_*SD*_ = 0.03 vs. *M*_*control*_ = 0.22, *SE*_*control*_ = 0.02, t(94) = 3.82, *p* < 0.001, d = 0.78, 95% CI [−0.18, −0.06]; highly competent advisor: *M*_*SD*_ = 0.53, *SE*_*SD*_ = 0.02 vs. *M*_*control*_ = 0.47, *SE*_*control*_ = 0.03, t(94) = 1.55, *p* = 0.124, d = 0.32, 95% CI [−0.14, 0.02]) (see Fig. 1).

Chronotype was not related to advice taking. The self-reported chronotype did not correlate with WOA scores in neither in the SD condition, nor in the control condition (all *p*s > 0.3).

### Accuracy of estimates

First, we tested to see whether SD affected the accuracy of the estimates (independent of advice taking), and so we examined whether SD had an effect on the MAPE-scores of initial estimates. This analysis revealed that the initial estimates of the participants in the SD condition were slightly less accurate (*M* = 51.74, *SE* = 2.45), compared to the initial estimates of the participants in the control condition (*M* = 45.51, *SE* = 2.42), *t*(94) = 1.8, *p* = 0.074, d = 0.35, 95% CI [−0.13, .01].

To control for this baseline-difference for the following analyses regarding the effects of SD and competency of advisors on the accuracy of estimates, we calculated difference scores of MAPE-scores (ΔMAPE) of initial estimates and MAPE-scores of final estimates. Negative ΔMAPE indicate an improvement in accuracy, with higher negative values indicating stronger improvements. Next, we checked the intra-class correlation (ICC) for the baseline model. This model predicted ΔMAPE intercept (the grand mean of ΔMAPE) with a random intercept for trial and subject. The ICC for the ΔMAPE for subject was only 0.02, indicating very little variation is explained by random effects, suggesting a multi-level model as superfluous. Hence, we decided to conduct an ANOVA with repeated measures. A linear mixed effects analysis yielded the same pattern of results. We conducted a 2 (sleep deprivation: yes vs. no) ×2 (competency of advisor: medium vs. high) ANOVA with repeated measures on the second factor and ΔMAPE as dependent variable.

**Figure 2:**
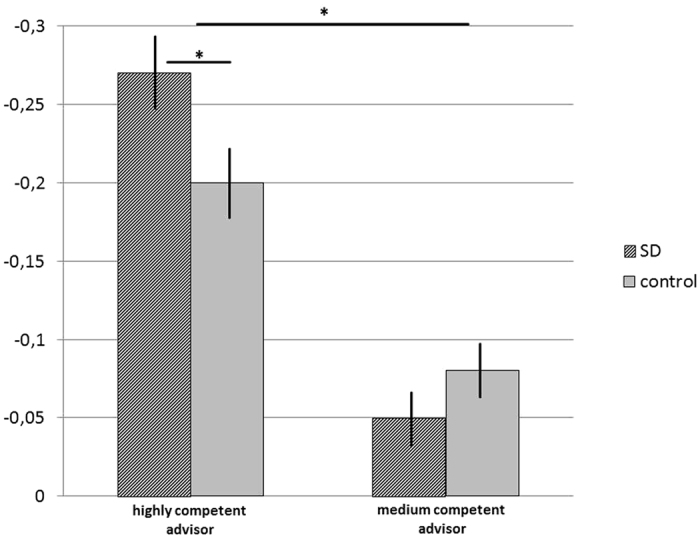
Improvements in MAPE scores from initial to final estimates by experimental condition in the highly competent advisor and in the medium competent advisor trials. Negative values indicate stronger improvements. Data presented as group means ± SEM. *p < 0.05.

This analysis revealed a main effect of competency of advisor, indicating that participants benefited more from the highly competent (*M* = −23.43, *SE* = 1.61), compared to the medium competent advisor (*M* = −6.46, *SE* = 1.2), *F*(1, 94) = 84.32, *p* < 0.001, ηp^2^ = 0.473, 95% CI [−0.21, −0.13]. In contrast, we found no significant main effect of SD on accuracy change, *F*(1, 94) = 0.64, *p* = 0.426, ηp^2^ = 0.007, 95% CI [−2.55, 5.99]. We found a two-way interaction of competency of advisor and experimental condition, *F*(1, 94) = 8.21, *p* = 0.005, ηp^2^ = 0.08. Disentangling the two-way interaction, we calculated the simple effects for trials with highly competent advisor and medium competent advisor with experimental condition as the independent variable. For the highly competent advisor trials, these analyses revealed a stronger improvement for the SD condition (*M* = −26.94, *SE* = 2.32) compared to the control condition (*M* = −19.92, *SE* = 2.23), *t*(94) = 2.18, *p* = 0.032, d = 0.44, 95% CI [0.01, 0.13]. In contrast, for the medium competent advisors, this comparison showed no statistical difference (SD condition: *M* = −4.67, *SE* = 1.72 vs. control condition: *M* = −8.25, *SE* = 1.65), *t*(94) = −1.5, *p* = 0.138, d = 0.25, 95% CI [−0.08, 01] (see Fig. 2).

A post-hoc test of judgmental accuracy of the final estimates (MAPE scores) in the highly competent advisor trials, showed no statistical difference between SD condition and control condition (*M*_*SD*_ = 22.98, *ME*_*SD*_ = 1.38; *M*_*control*_ = 23.57; *SE*_*control*_ = 1.51; *t*(94) = 0.29, *p* = 0.782, d = 0.06, 95% CI [−3.61, 4.78]).

### Mediation analysis

In an exploratory manner, we tested whether increased advice taking caused by SD resulted in increased improvements of judgment accuracy. Employing a bootstrapping procedure^36^ we tested an indirect effect of SD (independent variable) on ΔMAPE (dependent variable) via advice taking (mediator). As shown in Table 2, this analysis revealed an indirect effect: the 95%-bias-corrected bootstrap confidence interval (based on 10,000 bootstrap samples) for the indirect effect (*B* = −2.00) did not include zero [−4.19, −0.63].

## Discussion

We found that one night of total SD increased advice taking in an estimation task, compared to a control condition with regular sleep. In light of previous research showing that SD-induced impairments in cognitive abilities go hand in hand with decreased confidence^7^, we interpret our finding such that increased advice taking might have served as a coping strategy. Moreover, previous research has found higher susceptibility to social influence in sleep deprived individuals, presumably as a result of exhausted self-control resources^10^. Applying this idea to our findings, sleep deprived participants might have been more receptive to advice due to impairments in self-regulatory abilities^11^. Interestingly, the effect of SD on advice taking was particularly pronounced when the advisor was of medium competency. This finding indicates a tendency of sleep deprived individuals to rely more on decisional support, even if it is only of moderate quality. At first sight, this might be due to difficulties of sleep deprived individuals to differentiate between high and low quality information^4^. However, our study was designed to test whether SD has an effect on advice taking when the competency of advisor is known. Hence, in our study participants received rather unambiguous information of the competency of the two advisors (their rank position in a sample of 100 participants). And in each trial, they received information about which of the two advisors provided advice for that respective trial. Therefore, difficulties in correctly identifying the quality of advice are unlikely to apply as an explanation. In contrast, increased utilization of even moderate quality advice might reflect deliberative decisions by sleep-deprived individuals to more strongly rely on others. In any case, it would also be interesting to see future studies that whether SD has an effect on the correct perception of quality and source of advice.

Our analyses of judgmental accuracy revealed that sleep deprived participants’ initial estimates were slightly less accurate than the initial estimates of their rested counterparts. This decline in accuracy is likely to be a result of SD-induced impairments in basic cognitive abilities^6^,^24^. At the same time, sleep deprived participants were able to redeem this negative effect on judgmental accuracy due to increased advice taking – at least if advice was provided by a highly competent advisor. Hence, the good news is that although SD typically impairs decision making^4^, sleep deprived individuals are more open to taking advice which might even allow them to catch up with well-rested decision makers. Referring back to our example of sleep deprived politicians, our study emphasizes the importance for them to have access to highly competent and reliable advisors in times of reduced sleep.

### Limitations

Two limitations of our study have to be acknowledged. First, although the type of judge advisor systems used in our study – a quantitative estimation task, where judges received the estimations of unknown and non-present others - is very common in judge-advisor research^17^,^19^, there are drawbacks of this paradigm. It can be argued that merely presenting estimations by others is too broad an understanding of advice: It has been proposed that advice is information from others received in the form of an explicit recommendation or an opinion^37^. Therefore, it would be interesting to see if our findings replicate for decisional situations where advice is more directive. Bearing in mind the increased susceptibility to social influence in sleep deprived individuals^10^, we predict that the effects found in our study would be even stronger for more directive advice. For similar reasons, we hypothesize that with a physically present advisor the effects will be more pronounced: the presence of the advisor generates social pressure on the judge, by adding normative social influence (i.e., not ignoring advice so as to not insult the advisor) to the informational social influence^38^. Taking into consideration the rather small effect of SD on advice taking found in our study, creating conditions under which the effect of SD should be more pronounced (such as more directive advice from a physically present advisor) is definitely a fruitful avenue for future research. Particularly because such conditions would increase the ecological validity of experimental decision making situations and allow for better estimations of the real-life consequences of SD. We see our study as a starting point to investigate advice taking in the context of sleep deprivation. We therefore decided to employ a well-established paradigm of low complexity that allows for a clear-cut calculation of advice taking. We encourage future research to plough ahead using other more real-life judgmental tasks.

Second, our goal was to examine the basic effect of SD on advice taking. We did not employ measures to uncover potential underlying mechanisms, such as judgmental confidence or self-control strength. Because our study tapped new ground, and since this type of research is very labor-intense and costly, we felt it would be more adequate to start with a clear-cut test of the basic effect, rather than including additional measures that on their own are likely to affect performance on the task. Of course, we endorse future research that directly tests these possible causal paths for the effects of SD on increased advice taking.

## Conclusions

The main finding of our study is that one night of total SD increases advice taking in a judgmental task – particularly when advice is of only medium quality. Our study provides new insights into the widely neglected social effects of SD, such as mutual decision making and susceptibility to social influences. Furthermore, our study highlights the importance of studying and understanding how everyday factors, like sleep deprivation can influence decision making in social contexts.

## Additional Information

**How to cite this article**: Häusser, J. A. *et al.* Sleep Deprivation and Advice Taking. *Sci. Rep.* **6**, 24386; doi: 10.1038/srep24386 (2016).

## Figures and Tables

**Table 1:** Results of mixed effect modeling for Weight of advice (WOA scores).

Model	Random effects	Std.Dev	Fixed effects	Est.	Std.Err.	Df	T	p
0	Subject	0.14	Intercept	0.39	0.03	51.21	14.16	<0.001
	Trial	0.12						
	Residual	0.31						
1	Subject	0.13	Intercept	0.34	0.03	70.51	11.29	<0.001
	Trial	0.12	Condition	0.09	0.03	94.03	3.12	0.002
	Residual	0.31						
2	Subject	0.13	Intercept	0.23	0.03	96.42	9.23	<0.001
	Trial	0.05	Condition	0.09	0.03	94.03	3.11	0.002
	Residual	0.31	Advisor Competence	0.22	0.02	28.04	9.71	<0.001
3	Subject	0.13	Intercept	0.22	0.03	106	8.46	<0.001
	Trial	0.05	Condition	0.12	0.03	125.6	3.85	<0.001
	Residual	0.31	Advisor Competence	0.25	0.03	43.1	9.86	<0.001
			Condition: Advisor Competence	−0.06	0.02	2741.2	−2.59	0.0097

**Table 2:** Mediation model for the indirect effect of experimental condition on accuracy.

Predictor		Outcome						
		WOA-values (M)				MAPE difference (DV)		
		*B*	*SE*	*t*		*B*	*SE*	*T*
Experimental condition (IV)	*a*	0.091	0.03	3.12**	*c*	−1.72	2.15	−0.8
					*c*′	0.28	2.17	0.13
WOA-values (M)					*b*	−21.96	7.29	−3.01**
Indirect						−2.00	0.92	−2.19*
						*R*^2^ = 0.095		
						
						*F*(2, 93) = 4.89*p* = 0.01		

Note. N = *96.* Unstandardized regression coefficients are reported. Experimental conditions are dummy-coded: 0 = control condition, 1 = SD condition. *p < 0.05 **p < 0.005.
